# Variation in human 3D trunk shape and its functional implications in hominin evolution

**DOI:** 10.1038/s41598-022-15344-x

**Published:** 2022-07-11

**Authors:** Markus Bastir, José María González Ruíz, Javier Rueda, Gonzalo Garrido López, Marta Gómez-Recio, Benoit Beyer, Alejandro F. San Juan, Enrique Navarro

**Affiliations:** 1 Paleoanthropology Group, Museo Nacional de Ciencias Naturales, CSIC, J.G. Abascal 2, 28006 Madrid, Spain; 2 Department of Health and Human Performance, Faculty of Physical Activity and Sports Sciences-INEF, Universidad Politécnica de Madrid, 28040 Madrid, Spain; 3 Laboratory of Functional Anatomy (LAF), Faculty of Motor Skills Sciences, Université Libre de Bruxelles, Brussels, Belgium

**Keywords:** Bioenergetics, Computer modelling, Biological anthropology, Respiration, 3-D reconstruction, Musculoskeletal system, Physiology, Anatomy, Evolution, Anthropology, Palaeontology

## Abstract

This study investigates the contribution of external trunk morphology and posture to running performance in an evolutionary framework. It has been proposed that the evolution from primitive to derived features of torso shape involved changes from a mediolaterally wider into a narrower, and antero-posteriorly deeper into a shallower, more lightly built external trunk configuration, possibly in relation to habitat-related changes in locomotor and running behaviour. In this context we produced experimental data to address the hypothesis that medio-laterally narrow and antero-posteriorly shallow torso morphologies favour endurance running capacities. We used 3D geometric morphometrics to relate external 3D trunk shape of trained, young male volunteers (N = 27) to variation in running velocities during different workloads determined at 45–50%, 70% and 85% of heart rate reserve (HRR) and maximum velocity. Below 85% HRR no relationship existed between torso shape and running velocity. However, at 85% HRR and, more clearly, at maximum velocity, we found highly statistically significant relations between external torso shape and running performance. Among all trained subjects those with a relatively narrow, flat torso, a small thoracic kyphosis and a more pronounced lumbar lordosis achieved significantly higher running velocities. These results support the hypothesis that external trunk morphology relates to running performance. Low thoracic kyphosis with a flatter ribcage may affect positively respiratory biomechanics, while increased lordosis affects trunk posture and may be beneficial for lower limb biomechanics related to leg return. Assuming that running workload at 45–50% HRR occurs within aerobic metabolism, our results may imply that external torso shape is unrelated to the evolution of endurance running performance.

## Introduction

### Evolutionary anatomical changes

The trunk consists of the ribcage, the spine and the pelvis. During human body shape evolution, each of these elements experienced specific morphological changes. For example, the ribcages of *Homo erectus* and Neandertals were not only wider at the level of the central and lower thorax, but also antero-posteriorly deeper than most modern human populations^1–4^. Also the pelvis shows a systemic evolutionary trend towards reduction of its bi-iliac width, when comparing modern humans with *H. erectus* and members of the Neandertal lineage^5–8^. Evolutionary changes in the spine of the genus *Homo* show changes in overall height, it’s position within the ribcage and possibly spine curvatures. Within *Homo*, the overall spine length has increased, as a consequence of larger body size^9^. Greater dorsal orientation of the transverse processes in non-modern humans likely positioned the thoracic vertebral bodies more within the ribcage, producing a greater spine invagination^10,11^. Also, in Neandertals a smaller lumbar lordosis (hypolordosis) is discussed and could be particularly relevant with respect to trunk morphology as it directly affects the position and orientation of the sacrum and, thus, the pelvis^12–14^. The potential adaptive significance and functional implications of these features in hominin trunk evolution are not well understood and have been discussed in the context of thermo-regulatory^15^, digestive^16^, respiratory^3^, and locomotor functions^17^. Here, we focus on the latter two aspects.

Trunks with a narrow lower thorax and a narrow, tall waist have been associated with emerging endurance running capacities, possibly appearing with African *H. erectus* and together with elongated lower limbs^18^. Yet, a recent reconstruction of the KNM-WT 15,000 African *H. erectus* ribcage seems more similar to Neandertals in terms of width and depth than to modern human populations^3^. Nevertheless, Neandertals are thought to show adaptations for sprinting based on the anatomy of their foot skeleton^19^, and for power locomotion, as paleoecological and genetic evidence indicates, which is interpreted in the context of ambush hunting in a forested ecosystem^20^.

Thus, given the new evidence for greater similarities of trunk shape in primitive *Homo* and Neandertals^3^, together with known differences in the lower limb anatomy,—i.e. longer limbs in *H. erectus* adapted to endurance running^17,18,21,22^, and shorter limbs with specialized feet in Neandertals adapted to sprinting^19,20,23^—it is interesting to investigate the implications of variation in trunk morphology in the context of locomotor capacities.

### Trunk anatomy and running capacities

The trunk contributes to locomotor performance and energetics in two different ways: (1) the effect of trunk morphology on limb biomechanics, and (2) the effect of thorax morphology on breathing mechanics. Grossly speaking, sprinting and endurance running differ at energetic and locomotor (limb) biomechanics in the context of stride lengths, frequency and energetics. It has been shown that runners with relative longer lower limbs have lower locomotor costs^24^. Effective sprinting requires greater stride length^25^ and powerful lumbar muscles, specifically erector spinae and quadratus lumborum^26^. Endurance running, nevertheless, does not require longer strides. Higher frequency is more important to running performance during long distances and time, especially in longer trails, where the loss of stride length typically appeared due to fatigue^27,28^. Generally, a more upright trunk posture is observed among runners who perform efficiently in comparison with those less efficient, whose trunks were increasingly flexed during endurance running^29^.

Besides a positive effect of overall trunk muscularity^26,30^ on running performance, it has been shown that several other specific trunk morphological aspects relate to running performance, including the width of the pelvis^8,31^, the trunk flexion angle^32^, lumbar lordosis^33,34^ and associated hip flexion^31^, and thorax breathing mechanics^35,36^.

Within modern humans, the relationship between the widths of the thorax and the pelvis are important parameters of human variability in form and function^37^. The narrower pelvis relative to the wider thorax in males is associated with a gait pattern that differs biomechanically from that of females, who are characterized by a wider pelvis and narrower thorax dimensions^38^. The width of the pelvis influences the biomechanics of the psoas major affecting its hip rotator and flexor capacities^31^. Trunk flexion also affects significantly stride kinematics and kinetics. Although the factors of trunk flexion are unclear, higher trunk flexion angle correlates with shorter stride length, higher stride frequency, greater reaction forces and increased locomotion costs^32^.

Lumbar lordosis varies considerably in human populations^12,39–41^ and affects locomotor capacities. It has been shown that greater lordosis facilitates shock absorption, for example, when running^34^, while weaker lordosis produces a more forwards orientation of the pelvis, which is beneficial for leg return during sprinting^31^. Weaker lordosis is also related to greater trunk muscle strength^33^. Overall trunk muscularity (e.g. erector spinae, quadratus lumborum, psoas major, transverse abdominal, etc.…) has been correlated positively with sprinting capacities^26,30^. Differences in the tonus of the erector spinae and quadratus lumborum muscles have been related to greater lumbar lordosis^42^.

The contribution of thorax shape to trunk morphology is further interesting in the context of respiratory biomechanics^35,36^. It has been suggested that morphological features of the rib joints are relevant for ventilatory capacity during running^43^. These authors showed that *H. erectus* has similar rib joint morphologies as modern humans that differed from Australopithecus and chimpanzees. But also overall thorax morphology is important: antero-posteriorly flatter ribcages with more inferiorly declined ribs were suggested to show different thoraco-diaphragmatic and abdominal muscle recruitment during ventilatory movement than antero-posteriorly deeper thoraces with more horizontally aligned ribs^44–46^. Although pump- and bucket-handle patterns of rib motion seem more uniformly distributed along the ribs than originally assumed^47,48^, variation in thorax-shape related breathing biomechanics indirectly affect the locomotor capacities due to energetic competition and demands between the locomotor and the respiratory systems^49,50^. Thus, several studies have so far addressed the implication of specific elements of trunk morphology in isolation on locomotor performance. This study explores the relationship between entire 3D trunk shape and running performance based on virtual and geometric morphometric methods^51,52^. In the light of the functional anatomical evidence reviewed above, we address the hypothesis that trunks with an antero-posteriorly flat ribcage, a medio-laterally narrow pelvis and a lower lumbar lordosis are associated to a better running performance.

## Materials and methods

### Functional analyses, variables and experimental set up, ethics

Twenty-seven healthy trained young male students of the Degree in Sciences of Physical Activity and Sports (Table 1) were voluntarily recruited. Twelve of them were trained in endurance (ER) disciplines and fifteen were team sport players (non-ER). The inclusion criteria were the following: (1) Age between 18 and 30 years; (2) volunteers athletes had to be either long distance runners or team sports players (e.g., rugby, soccer, basketball); (3) not having suffered a musculoskeletal injury one month prior to the date of the protocol (i.e., checked through a previous exclusion questionnaire). And exclusion criteria were: (1) Age younger than 18 years; (2) having consumed any narcotic and/or psychotropic agents or drugs during the test; (3) any cardiovascular, metabolic, neurologic, pulmonary, or orthopaedic disorder that could limit performance in the different tests. Informed consent was obtained by all volunteers. The study protocol adhered to the declaration of Helsinki and was approved by the Ethics Committee of the Technical University of Madrid (Spain).

**Table 1:** Descriptives of the sample showing age, body size, and weight.

	Age (yr)	Stature (m)	Body weight (kg)	BMI
N	27	27	27	27
Min	18	1.62	53.50	19.97
Max	29	1.90	83.00	25.76
Mean	20.78	1.77	69.06	22.06
SD	2.53	0.07	7.20	1.34

All the participants performed a physiological (ramp) protocol on a treadmill (Telju JT4100-Liton -035, Toledo, Spain) in three different phases of exercise intensities: 45–50%, 70%, and 85% of the heart rate reserve (HRR). These three intensities correspond with the cardiorespiratory phase 1 [i.e., Light intensity, below the ventilatory threshold (VT)], phase 2 [i.e., Moderate intensity, between the VT and the respiratory compensation threshold (RCT)], and phase 3 [i.e., High intensity, above the RCT)]^27^. The rate of perceived exertion (RPE) was introduced as a complement of the HRR to help the control of the adequate intensity in each of the three submaximal workloads (i.e., For a HRR of 45–50% the RPE should be 2–4/10, HRR of 70% the RPE 5–6/10, and for HRR 85% the RPE ≥ 8/10). Before warm-up, rest heart rate was measured in sitting position until it was stable. After a general warm-up, the test started between 6 and 7 km h^−1^ and 1% of slope to mimic effects of air resistance^53,54^. Then, running velocity was increased by 0.5 km h^−1^ every 30 s until the achievement of HRR ≥ 85%, RPE ≥ 8 and volitional exhaustion. The following variables were recorded at these instances: time, running velocity, and RPE. Heart rate (beats·min^−1^) was continuously monitored during the test using a telemeter (Polar Ceinture H10+; Polar Electro OY, Kempele, Finland). Changes in velocity during the different work load phases were analysed by repeated measures ANOVA carried out in PAST^55^. Anthropometrical and running performance data were collected and summarized in Table 1 and Table 2.

### 3D shape data collection and geometric morphometric analyses

**Figure 1:**
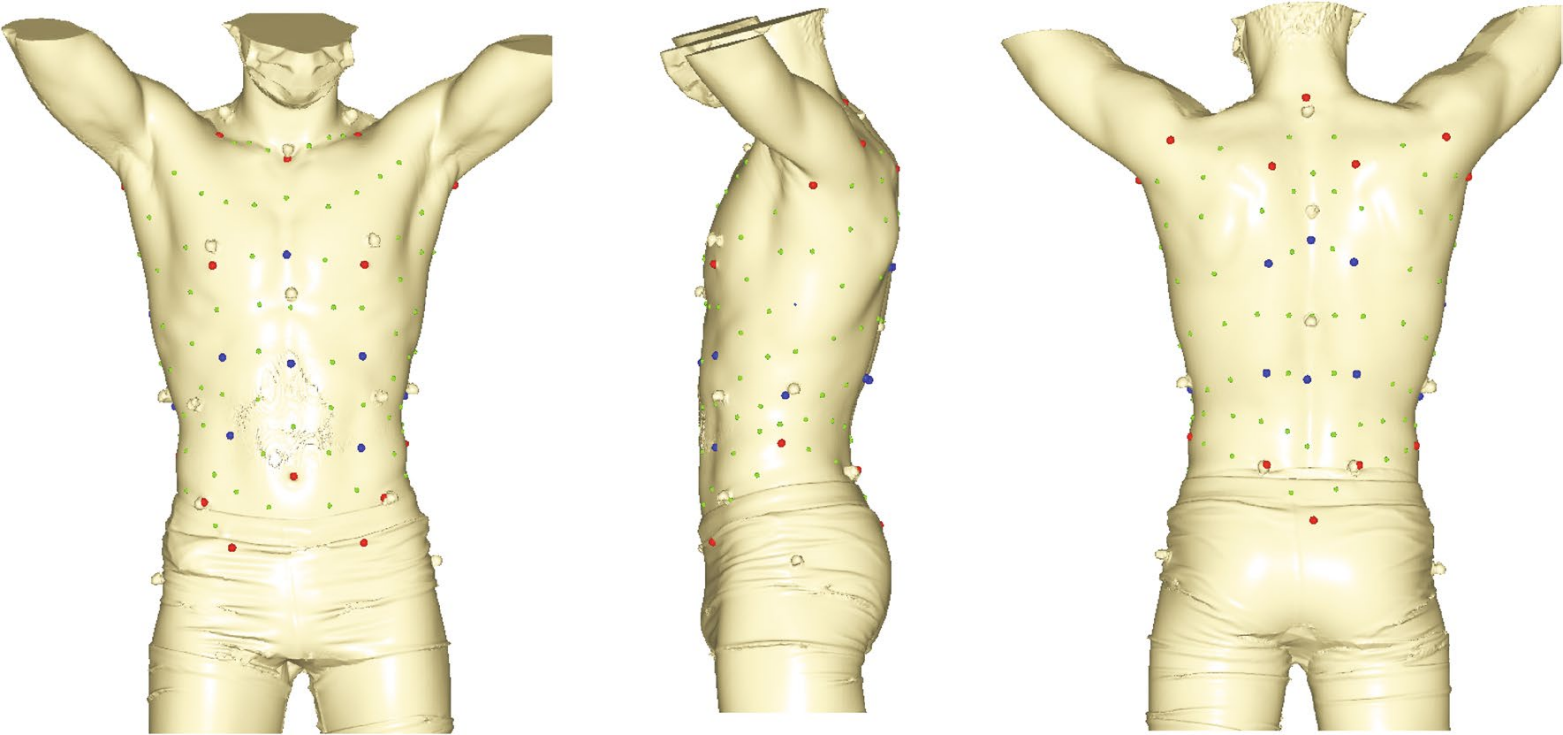
Frontal, lateral and posterior views of the 3D landmarks on the trunk surface. Red dots are fixed landmarks (Supplementary Table 1) and anatomically homologous between subjects, blue dots are curve semilandmarks, and green dots are surface semilandmarks. After resliding the semilandmarks are mathematically homologous among subjects.

3D body surface data were manually recorded by an Artec MHT 3D (www.artec3d.com) surface scanner in standardized positions, standing upright on a turning table, with quiet breathing and the arms slightly raised over the head to leave the 360° of the trunk contour free for image caption. 160 landmarks and semilandmarks (Fig. 1) were digitized according to the template described in González-Ruiz et al.^52^ (Supplementary Table 1), and postprocessed following standard methods^56^. Trunk landmark data were then analysed and visualized following standard methods of virtual, geometric morphometric analyses^51^. Specifically, generalized procrustes analysis (GPA) was carried out to obtain 3D shape data and multivariate regression analyses were carried out on the 3D shape data on running velocity. In order to account for different influence of muscularity on torso shape in endurance and non-endurance athletes, we performed a pooled-within group regression. We also tested the hypotheses with a reduced torso landmark set (N = 142 lms), where those landmarks that covered the skin surface related to the latissimus dorsi and pectoralis major muscles were removed. Finally, we explored the data for a possible impact of stature and weight on running performance using GLM. We set the significance level for the regression analyses on *p* < 0.05. The analyses were carried out using MorphoJ software^57^, PAST v3.25^55^, STATISTICA v.8^58^, geomorph package for R^59,60^ and Evan toolkit^61^ following the workflows outlined in Bastir et al.^51^ (Table 2).

**Table 2:** Descriptive statistics of the running velocities at different experimental steps.

	V_initial_ (km/h)	V1 (km/h)	V2 (km/h)	V3 (km/h)	V_max_ (km/h)
N	27	26	26	27	27
Min	6	6	8	12	12
Max	8	9	14	20	20
Mean	6.85	7.35	10.33	14.42	15.07
SD	0.43	0.75	1.35	2.09	1.81

## Results

**Figure 2:**
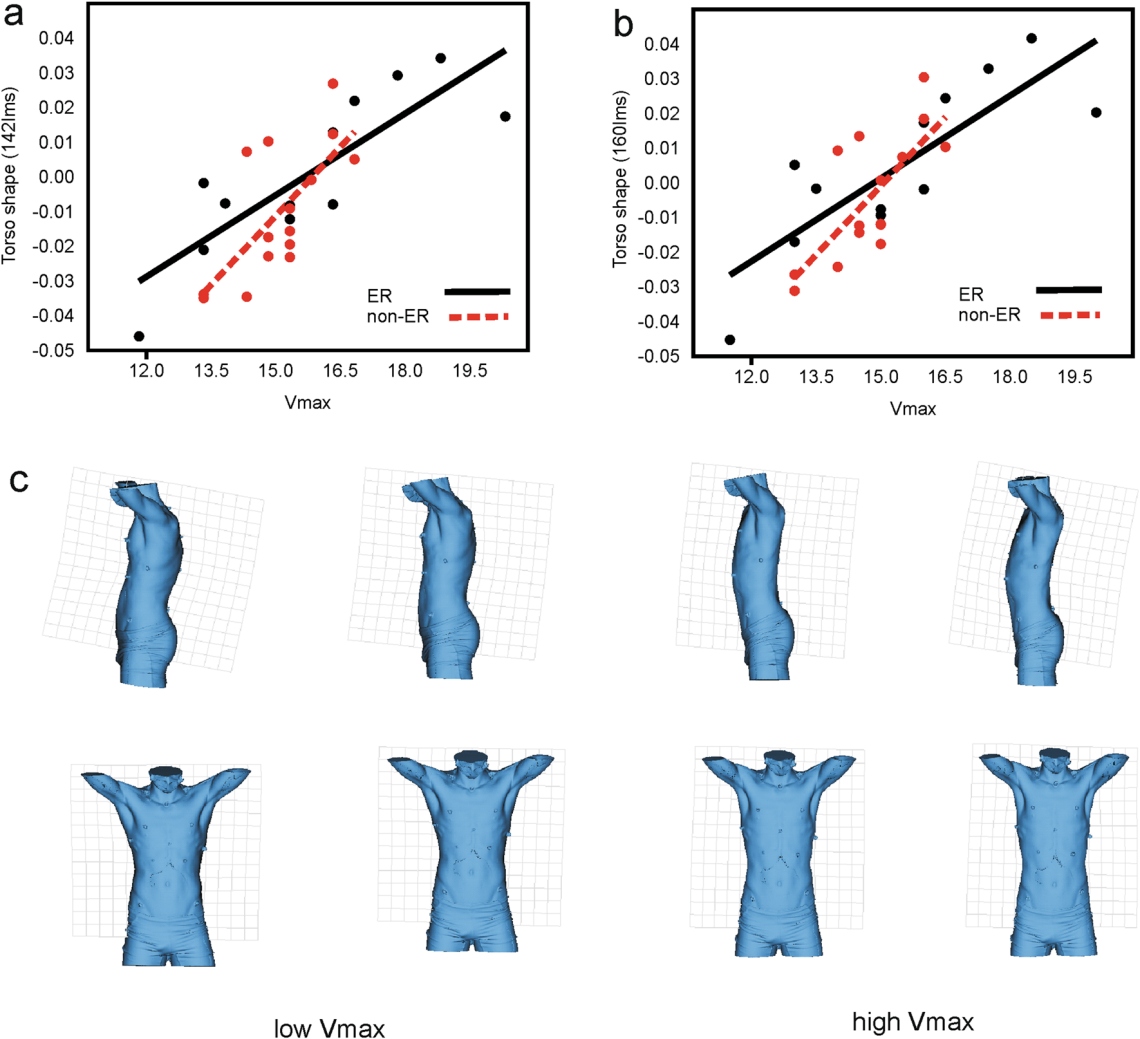
Torso shapes (160 lms) and thin-plate splines warped to the highest and lowest velocity at maximum intensity and running speed. (**a**) Non-muscular torso shape (142 lms) on maximum velocity. (**b**) Full torso shape (160 lms) on maximum velocity (**c**) Full torso shape warped to the configuration of lowest (left) and highest (right) maximum velocities. Upper panel left lateral view, lower panel frontal view. Note that flatter ribcages, narrower trunks with low thoracic kyphosis and more pronounced lumbar lordosis correlate significantly with higher velocities at maximum intensity. (Magnification factor from left to right: − 7.5; − 5; + 5, + 7.5, for better visualization).

Repeated-measures ANOVA (Table 3) shows that mean velocity increased significantly during incremental HRR phases (Fig. 2).

**Table 3:** ANOVA of velocities during the three different phases (V1, V2, V3).

	Sum of sqrs	df	Mean square	F	*p* (same)
Between groups	1526.05	4	381.51	393.5	< 0.001
Within groups	251.161	125	2.01		
Error	96.954	100	0.96		
Between subjects	154.207	25	6.17		
Total	1777.21	129

The regression analyses of torso shape on velocity phases indicated no significant relations during the first two stages (V1, V2) of workload. However, statistically significant relations were found between torso shape and velocity during phase 3 (V3) and maximum velocity (Vmax) (Table 4). Comparison of slopes in the ER and non-ER groups in the pooled within group regression models revealed no evidence for differences in the full torso shape data (P = 0.19; F = 1.833), nor in the non-muscular torso shape data (P = 0.18; F = 0.187) in relation Vmax. The GLM model revealed a significant influence of both, full and non-muscular torso shapes on running performance but no such effect of stature or weight (Table 5).

**Table 4:** Multivariate regressions of full torso shape (160lms) and non-muscular torso shape (142 lms) on running performance at different workloads (V1, V2, V3 and Vmax).

	Df	SS	MS	R^2^	F	Z	*p* Value
**160 lms**							
**V1**	1	0.003868	0.003868	0.04589	1.1543	0.54784	0.3
Residuals	24	0.080422	0.003351	0.95411			
Total	25	0.08429					
**V2**	1	0.00358	0.00358	0.04248	1.0646	0.30142	0.387
Residuals	24	0.08071	0.003363	0.95752			
Total	25	0.08429					
**V3**	1	0.005871	0.005871	0.06965	1.7968	1.8401	**0.034**
Residuals	24	0.078419	0.003267	0.93035			
Total	25	0.08429					
**Vmax**	1	0.007102	0.007102	0.08071	2.1948	2.4053	**0.009**
Residuals	25	0.08089	0.003236	0.91929			
Total	26	0.087991					
**142 lms**							
**V1**	1	0.003644	0.003645	0.04369	1.0965	0.39524	0.359
Residuals	24	0.079767	0.003324	0.95631			
Total	25	0.083412					
**V2**	1	0.003291	0.003291	0.03945	0.9857	0.082949	0.458
Residuals	24	0.080121	0.003338	0.96055			
Total	25	0.083412					
**V3**	1	0.005547	0.005547	0.0665	1.7096	1.728	**0.044**
Residuals	24	0.077865	0.003244	0.9335			
Total	25	0.083412					
**Vmax**	1	0.006828	0.006828	0.07836	2.1256	2.2905	**0.01**
Residuals	25	0.080303	0.003212	0.92164			
Total	26	0.08713					

(Note that sample size is N = 27 for Vmax, but N = 26 for V1, V2 and V3).

Significant values are in bold.

**Table 5:** Generalized Linear Models assessing the effects of stature, weight, torso shape (160 lms, 142 lms) on running performance.

	SS	df	MS	F	p
**160 lms**					
Intercept	7.93	1	7.93	5.38	**0.030**
Stature	0.51	1	0.51	0.35	0.562
Weight	0.88	1	0.88	0.60	0.447
Torso shape	46.85	1	46.85	31.79	**0.000**
Error	33.90	23	1.47		
**142 lms**					
Intercept	6.43	1.00	6.43	4.33	**0.048**
Stature	0.19	1.00	0.19	0.13	0.720
Weight	46.59	1.00	46.59	31.36	0.519
Torso shape	0.64	1.00	0.64	0.43	**0.000**
Error	34.16	23.00	1.49		

Significant values are in bold.

The associated 3D shapes (Fig. 2) show that the following morphological features of the trunk are positively associated with increased running performance: smaller antero-posterior diameter at the central-lower rib cage (flat thorax), narrower lower trunk (narrow pelvis), taller trunk, reduced thoracic kyphosis and more pronounced lumbar lordosis.

## Discussion

Modern humans are characterized by a relatively flat and narrow ribcage and pelvis when compared to fossil representatives of the genus *Homo* that are characterised by more stocky, wider and antero-posteriorly deeper torso configurations^2–6,15,62,63^. While more and more evidence seems to document this morphological trend, possible functional implications of reduced widths and depths of the trunk remain poorly understood. Because the trunk comprises elements of the respiratory and locomotor systems, the interaction of trunk shape with respiratory and locomotor performance is of specific interest.

In the present study, we address possible relations between torso shape and locomotor function in an experimental setting relating 3D external trunk surface shape with running velocity at different levels of intensity. The results showed no relationship between trunk shape and running performance at lower levels of exercise (V1, V2) below the anaerobic (respiratory) threshold, and just above it, indicating no relations between external torso shape and endurance running speeds between 7 and 10 km/h. However, at higher intensities and velocities above the anaerobic (respiratory) threshold (V3; average 14.4 km/h) a statistical relation between torso shape and running speed emerged. According to our results, subjects with a flatter and slightly narrower thorax, lower thoracic kyphosis, more pronounced lumbar lordosis, and slightly narrower pelvis can achieve such higher velocities such as indicated by the higher variances of 3D trunk shape shown at V3 and maximum velocity. It has been suggested that an endurance running velocity of about (5 ms^−1^ = 18 km/h) can be sustained by many amateurs without special training^18^, which is considerably faster than in our sample. At moderate intensity (V2), presumably within the aerobic metabolic domain, the average speed was about 10 km/h (Table 2). This may be related to the slight inclination of the treadmill (1%) during the incremental experiment (and the thereby simulated air resistance), but it could also reflect the fact that not all the volunteers were specialized endurance runners. Likewise, the average speed of 14 km/h at V3, which is likely already beyond the anaerobic threshold, is still lower than the published one and, again, could be related to the factors mentioned before. However, at and beyond this velocity, 3D torso shape was statistically related to running capacity.

The most visible features related to higher running capacities were a low degree of thoracic kyphosis, with a flatter, slightly narrower central thorax and a greater degree of lumbar spine curvature with a relatively slightly narrower pelvis. Covariation in depths was more clearly recognisable than in widths (Fig. 2). While the thoracic part suggests interpretation within a respiratory biomechanical perspective, the lumbo-pelvic part of the torso also requires consideration within functions of the locomotor system, although both are clearly related with each other. For example, the role of the posterior lumbar muscles is essential, as they act keeping an upright posture of the lower trunk during running and giving stability to the diaphragm and psoas major lumbar insertions. So, trunk extensors have the ability to reduce the kyphosis angle^64,65^. Links between breathing biomechanics and lumbar stability have been found in Kang et al.^66^ who showed that spinal posture was improved by specific breathing exercises in a clinical context.

The combination of a reduced thoracic kyphosis and a flat ribcage, with anteriorly declined ribs, in which the anterior rib ends are more caudally located than the posterior rib ends, could point to the importance of ventilatory biomechanics in higher intensity running. Bellemare et al.^44,45^ suggested that declined ribs can be elevated more during inspiration than horizontally aligned ones accentuating potentially the costal contribution to thorax movement during lung ventilation. Also, anteriorly declined ribs may have better biomechanical leverage during forced expiration, which crucially increases the tidal volume during heavy exercise breathing^35^. Because the declination of the ribs is morphologically related to a flatter rib cage configuration, the hypothesis that a flat thorax is positively related to running performance finds support. Physiologically, a less curved thoracic spine increases further the vertical space potentially available for lung expansion through enhancing of rib mobility. For example, negative consequences for lung ventilation due to kyphotic thoracic spine deformations, which compress thoracic space and affect rib biomechanics, have been reported^46,67^.

The implication of lumbar lordosis for locomotor biomechanics consists of its effect on the forwards orientation of the anterior superior iliac spine, which is an advantageous position for efficient leg return^31^. However, while these authors have not found a significant relation between lumbar lordosis angle and hip flexion capacity, our results in Fig. 2 clearly show that more pronounced lumbar curvature, to which also the lower thoracic kyphosis contributes, produces forwards tilt of the pelvis.

Warrener et al.^32^ have found a significant reduction of length and an increment of frequency of strides associated with higher trunk flexion posture during running. This finding is supported by Castillo and Liebermann^34^, who pointed out that higher lumbar lordosis (trunk extension) is linked with longer stride length in runners, a key factor in speed running as we have observed in our sample. Additionally, upright posture have been associated with better economy and running performance in the context mechanically interactions between trunk kinetics, reaction forces and spatiotemporal patterns of strides^29^.

Therefore, the empirical evidence reported in the present study seems to indicate that trunk evolution as a whole may have brought about the appearance of some features that are more clearly related to long distance running, along with others that are more related to power locomotion with higher workloads. However, these features lead to a mosaic notion, which reflects a complex picture of potential adaptations to running economy.

In Neandertals, some adaptations to power locomotion were proposed on anatomical, genetic, and ecological grounds^19,20^. Our results suggest that the relatively straight thoracic column along with their high level of trunk muscularity, possibly reflected by wide, deep thorax shape and associated high body mass estimates, would fit with the power locomotion hypothesis^2,68,69^. On the other hand, their supposed hypo-lordosis would argue against such interpretation as the relatively uncurved reconstruction of the thoracic and lumbar spine in the Kebara 2 Neandertal^13,69^ would indicate reduced pelvic tilt and thus a reduced capacity of leg return, hip flexion and sprinting capacity. Yet, the most recent reconstruction of the La Chapelle aux Saints Neandertal suggests vertebral curvatures similar to modern humans^14^ and this indicates that a better fossil documentation of lumbar spine anatomy in Neandertals is needed. Importantly, a recent study accounting for a wide range of population variability in modern humans, identified consistently and significantly more pronounced lordotic wedging in Neandertal L5 of Kebara 2, Shanidar 3, and La Chapelle aux Saints^41^ together with a more hypo-lordotic wedging in upper lumbar vertebra. Accordingly, this could suggest a completely different position of the lumbar spine within the trunk, with yet unclear biomechanical implications. Therefore, further fossil reconstructions of Neandertal torso skeletons together with experimental testing are necessary.

In African *H. erectus*, as reconstructed on the remains of KNM-WT 15,000, the straight thoracic^3^ and curved lumbar spine morphology^70^ would be more in line with effective power-locomotion. This, together with greater torso width and depth would be also compatible with higher muscularity and body mass^3,15,63,71,72^. However, clearly, the elongated limbs favour an interpretation of long-distance locomotion and, possibly, running^17,21^. Altogether, the present evidence and reviews suggest that our interpretations relate to a great extent on the reliability of the fossil body reconstructions.

However, it is important to bear in mind the limitations of our experimental evidence in the evolutionary context of endurance running. Obviously, the fossil record does not contain information about soft tissue anatomy, while the present data was exclusively collected on the external surface of the torso and so the relations between skeletal and soft tissue anatomy are unknown. Yet, bony features are considered. The curvature of the spine is assessed by the tips of the spinous processes which are variable in terms of sagittal orientations and thus do not directly inform about the curvature as assessable on the basis of the vertebral bodies. Also, the ribcage anatomy is only indirectly reflected by the skin surface landmarks and closer to skeletal thorax shape only at the central and lower parts of the rib cage. These data can thus only give a general idea about thorax shape. The pelvic landmarks are clearer in this respect as the iliac spines can be identified without problems. However, the reduced landmark set, which excluded shape information related to the latissimus dorsi and major pectoralis muscles may be less influenced by muscularity, and the fact that the results of the full and the reduced data are similar suggests little soft tissue effects on the results.

Further limitations are related to the proper running experiment. Endurance running in the evolutionary context appeared in the context of specific climatic conditions that were not considered in the present experiment. Also, actual endurance running is defined as running at intermediate velocities and aerobic conditions for longer time than considered in our experiment, where we only tested for potential relations between velocity and aerobic running conditions during the early stages of the incremental exercise. In this perspective, our data are only informative about shape-function relation during higher intensity running. Future studies should relate torso shape to running performance data on velocity and distance during longer trails and in hot weather conditions. Such analysis will provide further insight into the important relationships between torso shape, body shape and locomotor performance relevant for human evolution.

## Supplementary Information

Supplementary Information.
