# Supplementary material for: Variation in human 3D trunk shape and its functional implications in hominin evolution
Source: Sci Rep. 2022 Jul 11;12:11762. doi: 10.1038/s41598-022-15344-x (PMC9273616; doi:10.1038/s41598-022-15344-x)
Supplement: Supplementary file 1 — Supplementary Information. [file 41598_2022_15344_MOESM1_ESM.docx]

**Table 1. Anatomical locations of the 22 fixed landmarks. *landmarks in midsagittal plane.**

| Count | Landmarks |
| --- | --- |
| 1 | Right nipple |
| 2 | Right inguinal line |
| 3* | Top of the manubrium of the sternum |
| 4* | Belly button |
| 5 | Left nipple |
| 6 | Left inguinal line |
| 7 | Left armpit |
| 8 | Left lateral iliac crest |
| 9 | Left superior and medial angle of scapula |
| 10 | Left posterior and superior iliac spine |
| 11* | 7^th^ cervical spinous process |
| 12* | Intergluteal line |
| 13 | Right superior and medial angle of scapula |
| 14 | Right posterior and superior iliac spine |
| 15 | Right armpit |
| 16 | Right lateral iliac crest |
| 17 | Right acromial extremity |
| 18 | Left acromial extremity |
| 19 | Left lateral end of the spine of the scapula |
| 20 | Right lateral end of the spine of the scapula |
| 21 | Right anterior superior iliac spine |
| 22 | Left anterior superior iliac spine |
